# Self-assembly of 2D MnO_2_ nanosheets into high-purity aerogels with ultralow density†
†Electronic supplementary information (ESI) available. See DOI: 10.1039/c5sc03217b


**DOI:** 10.1039/c5sc03217b

**Published:** 2015-11-26

**Authors:** Zhenning Liu, Kongliang Xu, Ping She, Shengyan Yin, Xuedong Zhu, Hang Sun

**Affiliations:** a Key Laboratory of Bionic Engineering (Ministry of Education) , College of Biological and Agricultural Engineering , Jilin University , Changchun , Jilin 130022 , P. R. China . Email: sunhang@jlu.edu.cn; b State Key Laboratory on Integrated Optoelectronics , College of Electronic Science and Engineering , Jilin University , Changchun , Jilin 130012 , P. R. China

## Abstract

Self-assembling inorganic nanoparticles (NPs) into macroscopic three dimensional (3D) architectures often requires the assistance of organic components, leaving residual organics in the resultant. In this work, organic-free MnO_2_ aerogels with ultralow density have been achieved by the self-assembly of two dimensional (2D) MnO_2_ nanosheets *via* an ice-templating approach. To the authors' best knowledge, it is the first reported case of constructing a high-purity inorganic aerogel from preformed NPs without using any functionalization or stabilization agents. Moreover, it has been demonstrated that an ultralight MnO_2_ aerogel with a density as low as ∼0.53 mg cm^–3^, which is the lightest metal oxide aerogel to date, can be well obtained by such an approach. The successful formation of the aerogel can be attributed to the enhanced van der Waals force between the 2D building blocks that have been more orderly arranged by the squeezing of ice crystals during the freezing process. Hence, this work shows a pioneering example of assembling inorganic NPs into aerogels relying only on the weak interactions between NPs (*e.g.* van der Waals forces). It has also been demonstrated that the obtained MnO_2_ aerogel can function as an effective absorbent for toxic reducing gas, owing to its strong oxidation ability and high porosity. The strategy presented herein holds good potential to be applied to the fabrication of other high-purity inorganic aerogels, especially those with 2D building blocks readily available.

## Abstract 2

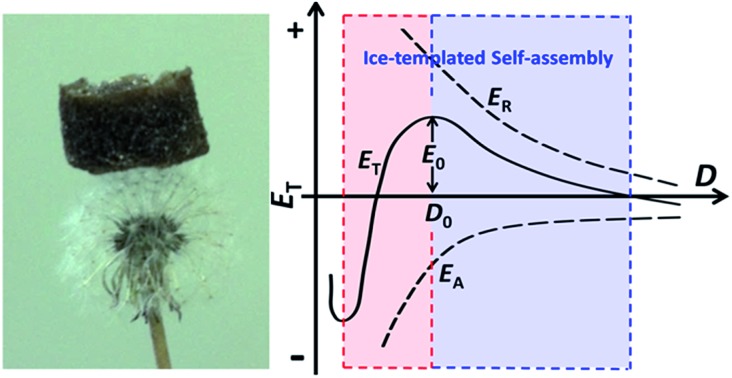
Organic-free MnO_2_ aerogels with ultralow density have been achieved by self-assembly of two dimensional MnO_2_ nanosheets *via* an ice-templating approach.

## Introduction

Self-assembling inorganic nanoparticles (NPs) into macroscopic three dimensional (3D) architectures is an important bottom-up strategy in nano-research, bridging the gap between individual NPs and structures suitable for practical applications.^1^–^3^ Aerogels are a class of 3D porous architectures with low density, large open pores and high surface area,^4^–^7^ which imparts them with a broad range of applications including thermal insulation, toxicant absorption, energy conversion and storage, catalyst support, *etc.*^8^–^16^ Assembly of inorganic (*e.g.* metal, oxide, chalcogenide, or pnictide) NPs into such functional aerogels has attracted broad attention in recent years.^17^–^21^ The resultant aerogels usually exhibit hierarchical structures with controlled crystallinity and composition, which can afford the chemical and physical features of NPs in addition to typical properties of macro-porous materials, and often integrate various properties in one material.

A common strategy of organizing NPs into a 3D percolating network is to install organic functionalities onto the surface of inorganic NPs to effect cross-linking. For instance, organics, such as 2-[2-(2-methoxyethoxy)ethoxy]acetic acid, polyethylenimine, sodium dodecyl benzene sulfonate (SDBS), sodium cholate (SC), *etc.* have been utilized to assist aerogel formation from preformed inorganic NPs.^18^,^22^,^23^ Unfortunately, since the assembling force provided by these exogenous organic components is critical to the construction of such aerogels, the obtained aerogels are indeed a hybrid of organic and inorganic materials and often incur unwanted property impairment caused by the cross-linkers. Alternatively, controlled destabilization of NPs in dispersion (*e.g.* partial removal of the surface stabilizing agent by oxidation) has also been adopted to induce self-assembly of NPs into inorganic aerogels, such as the aerogels of chalcogenide,^19^ pnictide,^17^ noble metal,^24^,^25^ *etc.* Nonetheless, the aerogels formed by destabilization normally contain trace amounts of impurities as a consequence of an incomplete removal of the surface stabilizing agents and suffer from reduced performance (*e.g.* charge transport and thermal stability) due to the residual organics.^24^ Hence, an approach to fabricate high-purity inorganic aerogels from preformed NPs is still in great need.

Ice-templating is an inexpensive, convenient and scalable technique that exploits endogenous ice crystals as templates to shape and press building blocks to achieve a desired structure.^26^–^28^ Recently, aerogels of Ag, Cu and MnO_2_ have been constructed by ice-templating from polyvinylpyrrolidone (PVP)-stabilized Ag/Cu nanowires and dimethylformamide (DMF)-capped MnO_2_ nanoflowers, respectively.^29^–^31^ Yet, the assembly of NPs in these aerogels still, at least partially, relies on the interaction of organic assisting agents. Therefore, a genuine case of assembling high-purity aerogels from inorganic-only building blocks is still lacking.

In recent years, two-dimensional (2D) nanosheets, such as MnO_2_, MoS_2_, BN, and so forth, have emerged as interesting functional materials,^32^–^40^ exhibiting large specific areas and strong self-assembly as a result of van der Waals forces, hydrogen bonding, *etc.*, and thus, have offered promising starting building blocks to assemble pure inorganic aerogels. However, due to the difficulty and/or limited options of manipulating inorganic interactions,^1^,^41^ it remains a grand challenge to control inorganic assembling processes and force these nanosheets into desired 3D structures.

In this contribution, we have successfully self-assembled a high-purity inorganic aerogel *via* ice-templating starting with monodispersed organic-free 2D MnO_2_ nanosheets. To the best of our knowledge, it is the first reported case of achieving a pure inorganic aerogel from preformed NPs without using any functionalization or stabilization agents. The resultant MnO_2_ aerogels also show extremely low density (as low as ∼0.53 mg cm^–3^), indicating the lightest metal oxide aerogels to date. The successful formation of the aerogel can be attributed to the enhanced van der Waals force between the 2D building blocks that have been more orderly arranged by the squeezing of ice crystals during the freezing process, demonstrating a novel approach to construct inorganic aerogels by only relying on weak interactions. It has also been shown that the obtained MnO_2_ aerogel can function as an effective absorbent for toxic reducing gas, owing to its strong oxidation ability and high porosity. The strategy presented herein has good potential to be applied to the fabrication of other high-purity inorganic aerogels, especially for those with 2D building blocks readily available.

## Results and discussion

**Fig. 1:**
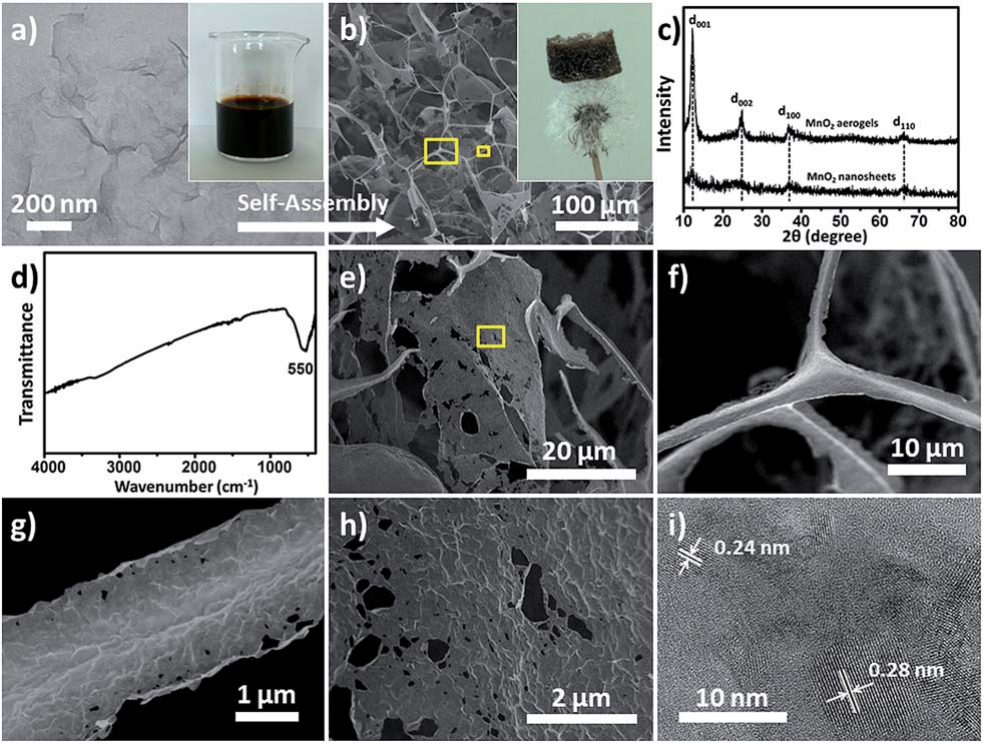
(a) TEM image and photograph (inset) of the colloidal MnO_2_ nanosheets; (b) SEM image of the obtained MnO_2_ aerogel and photograph of the aerogel standing on a dandelion (inset); (c) XRD profiles of the MnO_2_ aerogels (top) and MnO_2_ nanosheets (bottom); (d) FT-IR spectrum of the MnO_2_ aerogels; (e–h) SEM images of the MnO_2_ aerogel showing different micro-morphology: (e) a micro-scale 2D flake; (f) a trident node (the large box in (b)) at higher magnification; (g) a 1D rod (the small box in (b)) at higher magnification; (h) a local area of the 2D flake (the box in (e)) at higher magnification; (i) high-resolution TEM image of the aerogel.

High-purity MnO_2_ aerogels were self-assembled from monodispersed MnO_2_ nanosheets by fully manipulating the interaction between inorganic NPs *via* an ice-templating approach. Building blocks of an MnO_2_ nanosheet, which displayed a 2D morphology with a lateral dimension mainly in the range of 170 to 240 nm and a typical thickness of 2–4 nm (Fig. 1a, S4 and S5†), were obtained by ultrasound-exfoliation of purified layered MnO_2_ nanosheets following our reported method with minor modification (see S2.1† for details).^42^,^43^ Then a colloid of organic-free building blocks (inset of Fig. 1a) was cultivated at a temperature (–20 °C) below the freezing point to form a 3D network as confined by the growing ice crystals. A free-standing cylindrical MnO_2_ aerogel can be obtained by the sublimation of ice in a freeze-dryer, which is light enough to stand on a dandelion without damaging it (inset of Fig. 1b). The obtained aerogel exhibits a biomimetic foam structure with interconnected macro-pores that can be distinguished by the naked eye. Subsequent measurement shows a density of around 1.0 mg cm^–3^ (*i.e.* 4.4 mg in 4.4 cm^–3^, Fig. S7†) and a high porosity of ∼99.9%, indicating the successful formation of an ultralight material (*ρ* < 10 mg cm^–3^).^44^,^45^ The microstructure of the aerogel has been further characterized by scanning electron microscopy (SEM), which reveals a 3D percolating network with open pores ranging from hundreds of nanometres to tens of micrometres (Fig. 1b), confirming the self-assembly from nanoscale into macroscopic structure.

The X-ray diffraction (XRD) peaks of the MnO_2_ building blocks (bottom curve in Fig. 1c) can be indexed to δ-MnO_2_ (JCPDS no. 18-0802) and the broad and low intensity XRD peaks indicate a poor crystalline or polycrystalline feature.^46^ The self-assembled aerogel (upper curve in Fig. 1c) shows characteristic peaks (d_001_, d_002_, d_100_ and d_110_) at the same positions as those of the starting MnO_2_ nanosheets. It should be noted that the interlayer peaks (corresponding to d_001_ and d_002_) of the aerogel are sharper and stronger, suggesting higher crystallinity of the MnO_2_ assembly and a more ordered arrangement of the nanosheets as conferred by ice-templating.^10^,^47^,^48^

The Fourier transform infrared (FT-IR) spectrum of the aerogel (Fig. 1d) only exhibits an evident band at 550 cm^–1^, which can be assigned to the vibrations of an octahedral [MnO_6_] framework, and no characteristic bands of organic compounds can be identified. Corroboratively, the elemental analysis of the MnO_2_ nanosheets (see S2.2† for details) shows no potential presence of organic elements (C and S), whereas only trace amounts of Na and K, representing inherent balancing cations, have been detected in addition to Mn and H. It has been found from the literature that reported inorganic aerogels assembled from preformed NPs normally require organic components to assist the assembly, and usually contain organic residues as a result (Table 1). Thus, to the best of our knowledge, this is the first reported case of achieving organic-free inorganic aerogels from preformed NPs without using any functionalization or stabilization agents.

**Table 1:** Comparison of purity and assembling force for recently reported aerogels assembled from preformed inorganic NPs

Reported aerogels	Preformed building blocks	Organic additives	Methods	Assembling force		Impurities	Ref.
BaTiO_3_ aerogels	BaTiO_3_ nanoparticles	2-[2-(2-Methoxyethoxy) ethoxy] acetic acid	Organic functionalized gelation	Organic interaction	Hydrogen bonding between the acetic acid	Acetic acid residue	18
Si/Ag/MnO_2_ aerogels	Si/Ag/MnO_2_ nanowires	Sodium dodecyl benzene sulfonate (SDBS)	Condensation assisted gelation		van der Waals force between SDBS	SDBS residue	23
MoS_2_/BN aerogels	MoS_2_/BN nanosheets	Sodium cholate (SC)	Condensation assisted gelation		van der Waals force between SC	SC residue	23
Ag/Cu aerogels	Ag/Cu nanowires	Polyvinylpyrrolidone (PVP)	Ice-templated assembly		van der Waals force between PVP	PVP residue	29 and 31
MnO_2_ aerogels	MnO_2_ nanoflowers	Dimethylformamide (DMF)	Ice-templated assembly		van der Waals force & hydrogen bonding between DMF	DMF residue	30
Ag hydrogels/aerogels	Ag nanoshells	Thiolate (glutathione)	Oxidative aggregation	Inorganic interaction	Fuse together between Ag	Thiolate residue	24
Pnictide aerogels	Pnictide nanoparticles	11-Mercaptoundecanoic acid	Oxidative aggregation		Fuse together between pnictide	Thiolate residue	17
Chalcogenide aerogels	Metal chalcogenide nanoparticles	4-Fluorophenylthiolate	Oxidative aggregation		Fuse together between chalcogenide	Thiolate residue	20
MnO_2_ aerogels	Monodispersed MnO_2_ nanosheets	None	Ice-templated assembly		van der Waals force between MnO_2_ nanosheets	No organics detected	This work

The micro-morphology of the MnO_2_ aerogel was examined in more detail by SEM and transmission electron microscopy (TEM). It has been found that the 3D network of the aerogel consists of two major types of microstructure: one-dimensional (1D) rods and 2D flakes, as revealed in both the top-view and sectional-view SEM images (Fig. 1b and S8†). The average length of the 1D rods is estimated as ∼50 μm, whereas the 2D flakes show a lateral dimension of ∼50 μm (Fig. 1e and S9†). Many “Y-shaped” trident nodes, made of three joined 1D rods, can be found in the SEM images (Fig. 1f and S10†) and these 1D rods usually possess a prismatic shape with concaved sides (Fig. 1g and S10†). The SEM and TEM images of higher magnification show that both the 1D rods (Fig. 1g and S11a†) and 2D flakes (Fig. 1h and S11†) exhibit multiple wrinkles, presumably as a result of the stacked nanosheets. It is noteworthy that the perceived average lateral dimensions of the nanosheets in 1D rods and 2D flakes are ∼200 nm (Fig. S11†), which are in good agreement with the observed size for the building blocks of colloidal MnO_2_ nanosheets (Fig. 1a and S4†). Moreover, the high-resolution TEM image (Fig. 1i) reveals that the obtained MnO_2_ aerogels are polycrystalline and that the lattice fringes show a *d*-spacing of 0.24 nm and 0.28 nm, corresponding to the *d* values of the (100) and (110) planes of δ-MnO_2_, respectively. Therefore, the obtained 3D aerogel demonstrates a hierarchical structure, that is, the 3D network is constructed of 1D rods and 2D flakes, which have been assembled from MnO_2_ nanosheets.

**Fig. 2:**
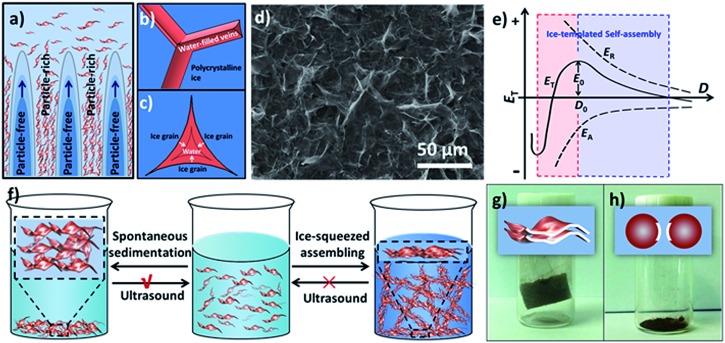
(a) Schematic diagram demonstrating the growth of ice crystals in the MnO_2_ colloid during freezing process; (b) schematic illustration of water-filled veins in polycrystalline ice, showing a “Y-shaped” trident node as a result of merging one vein into another at a junction of two veins; (c) cross-sectional illustration of a vein exhibiting a concave-faced prism shape; (d) SEM image of the sediments obtained from the freeze–thawed MnO_2_ colloid at 1.0 mg mL^–1^; (e) theoretical illustration of the relationship between the energy (*E*_T_, *E*_A_ and *E*_R_) and the distance (*D*) between NPs during ice-templated assembly; (f) schematic illustration for an ultrasound treatment of spontaneous sediments (left) and ice-squeezed assembly (right); (g and h) schematic (top) and photographic (bottom) illustrations of ice-templated assembly obtained from MnO_2_ nanosheets (g) and MnO_2_ nanospheres (h).

The concentrating and squeezing effect afforded by ice-templating has been proposed here as the major mechanism that controls the formation of the aerogel and determines the resultant morphology.^28^ When the liquid of the MnO_2_ colloids freezes below the freezing point, nucleation of ice crystals occurs randomly on the frozen surface of the colloid. Then, the ice nuclei gradually grow in the MnO_2_ colloids, eventually reaching a cellular morphology regime.^28^ MnO_2_ nanosheets, excluded from the ice crystals at the early stage of freezing, are repelled and concentrated by the growing ice crystals (Fig. 2a). Therefore, the solidifying body can be divided into two domains: particle-free regions, corresponding to the cellular ice crystals free of nanosheets, and particle-rich regions, corresponding to the concentrated nanosheets excluded by ice. Subsequently, the nanosheets in the particle-rich region start aggregating and form a 3D network confined by the growing ice crystals, which results in the microstructures of 1D rods and 2D flakes. This hypothetical mechanism can be supported by the findings of glaciology research. As revealed in the works of glaciology,^49^ an interconnected system of water-filled veins (Fig. 2b) can form in polycrystalline ice at a temperature near but below the freezing point. The veins lie along lines where three ice grains meet and are squeezed by these ice grains from three sides. As a consequence, the veins usually take on a shape of a curved-in prism (Fig. 2c), which may explain the concave prismatic morphology of 1D rods in this work (Fig. 1g). The junction of two veins usually merges one vein into another and results in a “Y-shaped” trident node (Fig. 2b), as observed by SEM (Fig. 1f). Similar observation has also been made by Chen's group for self-assembled Au NPs directed by polycrystalline ice.^50^ Furthermore, at the interface of two ice grains instead of three, 2D flakes (Fig. 1e) can be formed in the cleavage between the surfaces of two ice grains, which is also in line with the mechanism proposed by other groups.^10^,^31^

In order to verify that the microstructures of 1D rods and 2D flakes were formed by the concentrating and squeezing effect of ice during the freezing process, the icy chunk of the frozen colloid was thawed at room temperature and characterized by SEM. In contrast to the homogeneous colloid before freezing, the freeze–thawed sample shows colourless liquid with a large amount of brown sediment at the bottom (Fig. S14†), indicating that the nanosheets have aggregated during freezing. The SEM image of the sediment obtained from the freeze–thawed MnO_2_ colloid (1.0 mg mL^–1^) displays abundant 2D flakes accompanied by a few 1D rods (Fig. 2d and S15†), which is consistent with the SEM image of the aerogel (Fig. 1b). This observation suggests that the 1D rods and 2D flakes of the aerogel are indeed formed by the ice-templating effect on MnO_2_ nanosheets before the sublimation of ice, and that gentle ice removal (*i.e.* freeze-drying) is crucial to preserve the 3D network assembled in the freezing process.

Based on the Derjaguin–Landau–Verwey–Overbeek (DLVO) theory,^51^,^52^ the stability of NPs in solution is mainly determined by the balance between two factors, namely, repulsive force and attractive force. As shown in Fig. 2e, the total potential energy (*E*_T_) can be expressed as:

1 *E*_T_ = *E*_R_ + *E*_A_

where *E*_R_ is the repulsive energy (usually positive in value) and *E*_A_ is the attractive energy (usually negative in value).

Our MnO_2_ nanosheets demonstrate a zeta potential of around –10 mV and can form an aqueous colloid due to the electrostatic repulsion of the negative charge on their surfaces (Fig. S6†).^53^,^54^ When ice crystals nucleate and gradually grow in the colloid, the MnO_2_ nanosheets are squeezed into a closer proximity and the van der Waals forces between adjacent nanosheets increase, leading to a more negative value of *E*_A_ (Fig. 2e). Simultaneously, the repulsion between the negatively charged nanosheets will also elevate. However, the balancing cations (H_3_O^+^, Na^+^ and K^+^), also excluded and concentrated by the expanding ice crystals, can change the ionic atmosphere of the nanosheets and partially mitigate the rising repulsion between the negatively charged surfaces as they approach each other. As a result, although both the *E*_A_ and *E*_R_ increase in absolute value (*E*_A_ is more negative in value), as the distance (*D*) between the nanosheets decreases; *E*_A_ surges up in absolute value more quickly than *E*_R_. Therefore, whereas the increment in *E*_R_ dominates over *E*_A_ as ice crystals start to reduce *D*, the net incremental effect on *E*_T_ becomes zero at a certain critical point (*D*_0_), because the increment of *E*_R_ is neutralized by the increment of *E*_A_. As a consequence, once *E*_T_ reaches a maximum (*E*_0_) it begins self-declining, which further reduces *D* (*D* < *D*_0_) until steric hindrance stops this. At the macroscopic level, a stable assembly of nanosheets can be achieved as *E*_T_ declines.

It should be noted that the ice-squeezing effect plays a critical role in forming a stable assembly of 2D nanosheets, especially due to its rearrangement of the squeezed nanosheets. It has been found that the spontaneously formed sediments driven by gravity can be easily sonicated into a homogenous colloid by ultrasound-treatment (100 W) of a few seconds, whereas the freeze–thawed sediments from ice-squeezed assembly can not be redispersed into a colloid under the same ultrasound-treatment even after a longer period of 30 minutes (Fig. S14†), indicating the formation of a stable assembly. Such an observation is probably caused by two coordinated actions of squeezing ice (Fig. 2f). On one hand, growing ice crystals concentrate the nanosheets and balance cations as discussed above. On the other hand, the squeezing of ice crystals also aligns nanosheets in a more orderly fashion, as evidenced by the XRD profile of the aerogel (Fig. 1c), and leads to a larger effective surface for van der Waals interactions. As a consequence, the resultant ice-squeezed assembly requires more energy input than the spontaneously sedimented nanosheets to overcome a higher energy barrier to disassemble, and thus is more stable.

As a direct inference of the above explanation, the large effective surface for van der Waals attraction as afforded by 2D nanosheets is critical to the successful formation of the aerogel (Fig. 2g). To this end, MnO_2_ nanospheres with a smaller effective surface for van der Waals interaction were chosen as building blocks to construct aerogels under otherwise identical conditions as used for 2D MnO_2_ nanosheets (see S4.2† for details). After the removal of the ice, the 3D network of the frozen colloid collapsed into a powder instead of forming an aerogel (Fig. 2h and S17†), indicating that the van der Waals force between nanospheres is weak and cannot provide adequate strength to maintain the 3D architecture.

**Fig. 3:**
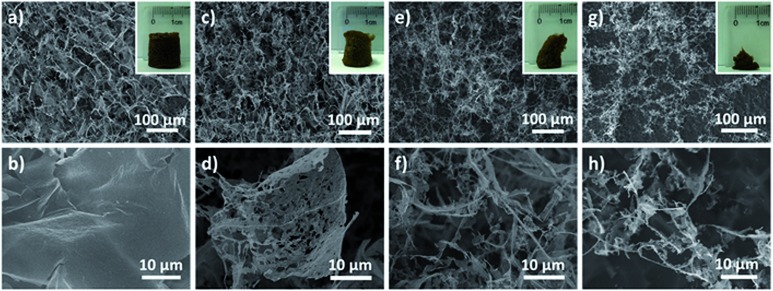
Top-view SEM images of MnO_2_ aerogels prepared from nanosheet colloids of different concentrations: (a and b) 1.0 mg mL^–1^, (c and d) 0.5 mg mL^–1^, (e and f) 0.25 mg mL^–1^ and (g and h) 0.1 mg mL^–1^. The inset images in (a), (c), (e) and (g) are the photographs of the corresponding aerogels. The bottom images of (b), (d), (f) and (h) are higher magnification images of (a), (c), (e) and (g), respectively.

Encouraged by the successful construction of an ultralight aerogel with a density of 1.0 mg cm^–3^, we set out to explore whether MnO_2_ aerogels with even lower density could be achieved. Aerogels were prepared from different concentrations of MnO_2_ nanosheet colloids (1.0, 0.5, 0.25 and 0.1 mg mL^–1^) in parallel, and then observed by naked eyes and SEM (Fig. 3). It has been found that the obtained MnO_2_ aerogels exhibit a trend of collapsing more with decreasing colloidal concentration (insets of Fig. 3a, c, e and g). Free-standing MnO_2_ aerogels of the container shape can be formed without evident collapsing at the concentrations of 1.0 and 0.5 mg mL^–1^ (insets of Fig. 3a and c), whereas the colloidal concentrations of 0.25 and 0.1 mg mL^–1^ result in obvious defects of the aerogel (insets of Fig. 3e and g). The density of the MnO_2_ aerogel constructed from the colloid of 0.5 mg mL^–1^ is estimated as ∼0.53 mg cm^–3^ (see S2.3† for the details of estimation method), which is the lowest reported density for metal oxide aerogels to the best of our knowledge. As a matter of fact, in the ultralight regime below 1 mg cm^–3^, only few materials are currently known: metallic microlattices (*ρ* ≥ 0.87 mg cm^–3^),^44^,^45^ aerographite (*ρ* ≥ 0.18 mg cm^–3^),^55^ graphene aerogels (*ρ* ≥ 0.16 mg cm^–3^),^14^ polyacrylonitrile/silica hybrid aerogels (*ρ* ≥ 0.12 mg cm^–3^),^56^*etc.* Our MnO_2_ aerogel (*ρ* ≥ 0.51 mg cm^–3^) adds the first member of metal oxide to the family of ultralight materials below 1 mg cm^–3^.

We next investigated the concentration dependence of the aerogel micro-morphology by SEM to elucidate the hierarchy of the 3D structure. It has been discovered that not only can more 2D flakes and less 1D rods be observed at higher concentrations of starting MnO_2_ colloid (Fig. 3b and d compared to Fig. 3f and h), but also less defects can be found in the 2D flakes formed from higher colloid concentrations (Fig. 3b compared to Fig. 3d). Although 1D rods can be viewed as extremely defected cases of 2D flakes, they coexist at a concentration even as low as 0.1 mg mL^–1^ (Fig. 3h), where the aerogel formed has demonstrated significant collapse. Hence, it has been proposed that during the formation of our aerogels, MnO_2_ nanosheets assemble into 1D rods and 2D flakes simultaneously, which are subsequently connected to form 3D compartmental networks. In addition, the fractions of 1D rods and 2D flakes can be tuned by altering the concentration of the starting nanosheet colloid. It is conceivable that below a critical concentration it is difficult for NPs to form sufficient 1D and 2D building blocks to maintain a well-shaped 3D network.

**Fig. 4:**
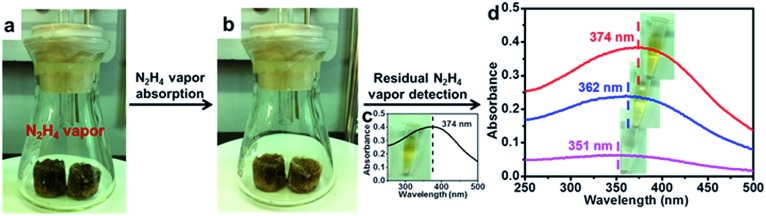
MnO_2_ aerogels as effective absorbents for N_2_H_4_ vapor. (a) Photograph of the pre-absorption MnO_2_ aerogels: two pieces of dark brown MnO_2_ aerogels were placed in the conical flask where N_2_H_4_ vapor would be produced by heating; (b) photograph of the MnO_2_ aerogels after hydrazine absorption showing that the color of the aerogel changed to yellow; (c) UV-vis spectrum and photograph (inset) of the colorimetric probe, which is 5 μg mL^–1^ colloidal suspension of MnO_2_ nanosheets as previously reported; (d) UV-vis spectra and photographs (inset) of the colorimetric probes after the detection of residual hydrazine: top curve (red) and inset are for the probe used in the detection of gas generated in the presence of MnO_2_ aerogels, middle curve (blue) and inset are for the probe used in the detection of gas generated in the presence of MnO_2_ powders, bottom curve (purple) and inset are for the probe used in the detection of gas generated in the absence of absorbents.

MnO_2_ is known for its oxidation ability and can be used as an absorbent for reducing toxicants.^57^ Thus, we set up a simple experiment to explore the potential to utilize our MnO_2_ aerogels to adsorb a toxic reducing gas. In particular, hydrazine (N_2_H_4_) was chosen for its easy evaporation from N_2_H_4_·H_2_O liquid at low temperature. The colour of MnO_2_ aerogels gradually changed from dark brown (Fig. 4a) to yellow (Fig. 4b) as the hydrazine gas was produced at 60 °C, indicating that the hydrazine vapour generated was indeed absorbed by the aerogel. The colour change of the aerogels can be attributed to the transformation of MnO_2_ into Mn(OH)_2_ by the reducing gas.^58^ A reported N_2_H_4_-sensitive colorimetric probe (Fig. 4c) was then prepared according to a previously reported method,^58^ and was used for the detection of residual hydrazine to verify the adsorption efficiency of MnO_2_ aerogels. No evident colour change of the colorimetric probe has be observed (top inset of Fig. 4d) and no significant change of absorption could be detected from the UV-vis spectrum (top line in Fig. 4d compared to pre-absorption curve in Fig. 4c), indicating that the hydrazine gas has been almost completely absorbed by the MnO_2_ aerogels. In contrast, in the absence of any absorbents, the yellow colour of the colorimetric probe quickly faded (bottom inset of Fig. 4d) and the absorption peak at 374 nm, the characteristic peak of the probe, was lost (bottom line in Fig. 4d). As a control, commercial MnO_2_ (c-MnO_2_) powders, have also been tested as absorbents under otherwise identical conditions. The colour of the colorimetric probe changed from yellow to light yellow (middle inset of Fig. 4d). Meanwhile, a peak shift (from 374 nm to 362 nm) and substantial decrease in absorption have been found in the UV-vis spectrum (middle line in Fig. 4d). Our MnO_2_ aerogels have demonstrated superior absorption efficiency over MnO_2_ powders, and are better candidates for applications in reducing gas absorption.

## Conclusions

In conclusion, it has been demonstrated that a high-purity inorganic aerogel can be assembled from 2D nanosheets *via* ice-templating without using any extra functionalization or cross-linking agents, which only relies on weak interactions between NPs. The MnO_2_ aerogels are simply prepared by freeze-drying the frozen colloids of 2D building blocks and the aerogels achieved exhibit a density as low as ∼0.53 mg cm^–1^, recruiting them as the first member of metal oxide in the ultralight material family (*ρ* < 1.0 mg cm^–3^). The resultant morphology and microstructures of the aerogel (*e.g.* 1D rods and 2D flakes) are in good consistency with the ice forming mechanism as determined in glaciology. The successful formation of the aerogel can be attributed to the enhanced van der Waals force between the 2D building blocks that have been more orderly arranged by the squeezing of ice crystals during the freezing process. It has also been demonstrated that the obtained MnO_2_ aerogel can function as an effective absorbent for toxic reducing gas, owing to its strong oxidation ability and high porosity. The ice-templating approach presented here provides a general strategy that holds good potential to be applied to the fabrication of other high-purity inorganic aerogels, especially those with 2D building blocks readily available.

## Supplementary Material

Supplementary information
