# Supplementary material for: Self-assembly of 2D MnO2 nanosheets into high-purity aerogels with ultralow density
Source: Chem Sci. 2015 Nov 26;7(3):1926–32. doi: 10.1039/c5sc03217b (PMC5966798; doi:10.1039/c5sc03217b)
Supplement: Supplementary file 1 [file SC-007-C5SC03217B-s001.pdf]

Electronic Supplementary Material (ESI) for Chemical Science.
This journal is © The Royal Society of Chemistry 2015

Electronic Supplementary Information (ESI)

Self-Assembly of 2D MnO_2 Nanosheets into High-Purity Aerogels with Ultralow Density

Zhenning Liu[a], Kongliang Xu[a], Ping She[a], Shengyan Yin[b], Xuedong Zhu[a], Hang Sun*[a]

[a] Key Laboratory of Bionic Engineering (Ministry of Education), College of Biological and Agricultural Engineering, Jilin University, Changchun, Jilin 130022, P. R. China.
E-mail: sunhang@jlu.edu.cn

[b] State Key Laboratory on Integrated Optoelectronics, College of Electronic Science and Engineering, Jilin University, Changchun, Jilin 130012, P. R. China.

S1. Materials and Characterization

S1.1Materials

$KMnO_4$ (A.R., Beijing Chemical Factory), sodium dodecyl sulfate (SDS, C.P., Tianjin Guangfu Fine Chemical Research Institute), concentrated hydrochloric acid (HCl, A.R., Beijing Chemical Factory), $Na_2S_2O_3$ (A.R., Fuchen Chemical Factory), hydrazine ($N_2H_4 \cdot H_2O$, A.R., Beijing Chemical Factory), commercial MnO_2 (c-MnO_2, A.R., Tianjin Huadong reagent factory), and alcohol (CH_3CH_2OH, A.R., Beijing Chemical Factory) were used as received. Carbon support film for TEM was purchased from Beijing XXBR Technology Co. All solutions were prepared using ultrapure water (resistance > 18 MΩ cm^{-1}).

S1.2 Characterization and Instruments

Transmission electron microscopy (TEM) images were obtained with a JEOL-2010 electron microscope operating at 200 kV. Scanning electron microscopy (SEM) images were collected on a JEOL JSM-7500F field emission scanning electron microscope. Atomic force microscopy (AFM) under tapping mode was carried out with a commercial instrument (Digital Instrument, Nanoscope III, and Dimension 3000) at room temperature in air. UV-Vis absorption spectra were taken in a quartz cell with light path of 1 cm on a SHIMADZU UV-

2500 spectrophotometer (200-800 nm). X-ray diffraction (XRD) was performed on a Rigaku X-ray diffractometer (D/max rA, using Cu Kα radiation at a wavelength of 1.542 Å), and the data were collected from 5 ° to 80 °. Fourier transform infrared (FT-IR) spectra were collected on an FT-IR spectrometer (SHIMADZU, IRAffinity-1) using KBr pellets (32 scans), and the spectra were recorded at a resolution of 4 cm^{-1}. Freeze-drying was carried in a freeze-dryer (Beijing Boyikang, FD-1C-50) at -50 °C. Elemental analysis for K, Na and Mn was carried on an ICP spectrometer (iCAP6300, Thermo Scientific) and elemental analysis for C, H, N and S on another elemental analyzer (Vario EI Cube, Elementar). A nitrogen adsorption isotherm at -196 °C was performed in an apparatus of Micromeritics ASAP 2020. The samples were degassed overnight at 300 °C before adsorption measurements and the specific surface area was calculated by using the BET equation.

S2. Preparation of the ultralight MnO_2 aerogels

S2.1 Synthesis of the layered MnO_2 nanosheets

Layered MnO_2 nanosheets were synthesized through a slow redox reaction between $KMnO_4$ and SDS as we previously described with minor modification. First, SDS solution (100 mM, 32 mL), HCl solution (100 mM, 3.2 mL), $KMnO_4$ solution (50 mM, 3.2 mL) and 281.6 mL ultrapure water were mixed together and heated at 95 °C for 3 hours. After heating, the solution became colorless (Fig. S1) and the MnO_2 nanosheets formed had precipitated. Then the precipitates were washed repeatedly with ultrapure water and alcohol to obtain pure MnO_2 nanosheets. The purified MnO_2 nanosheets were dried on corresponding substrates overnight at room temperature for characterizations of TEM, SEM and XRD.

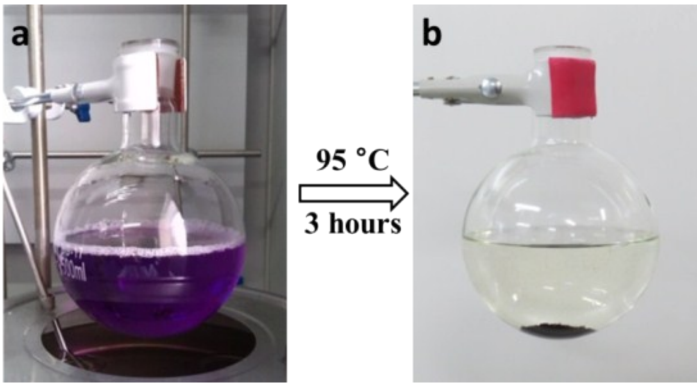

Fig. S1 a) photograph of the reaction mixture before heating; b) photograph of the reaction mixture after heating of 3 hours at 95 °C.

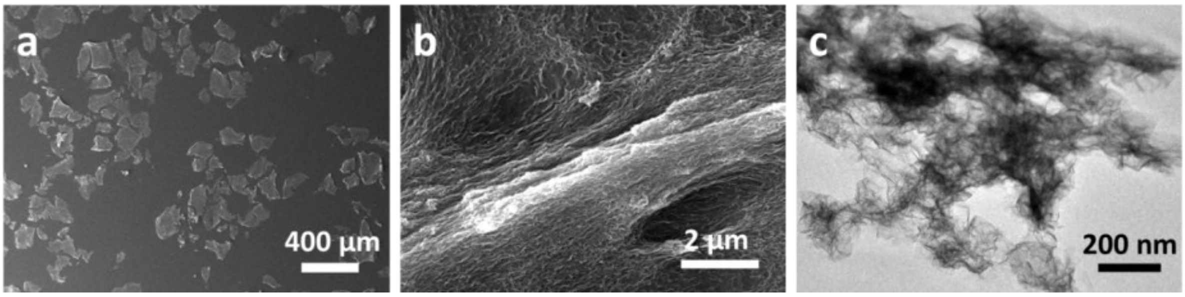

Fig. S2 a-b) SEM images of layered MnO_2 nanosheets on a silicon substrate at low (a) and high (b) magnifications; c) TEM image of layered MnO_2 nanosheets.

S2.2 Preparation of the monodipersed nanosheet colloid

The layered MnO_2 nanosheets, which have been formed by weak van der Waals interaction and stabilized by balancing cations (*e.g.* H_3O^+, Na^+ and K^+) intercalated between the MnO_2 layers, can be easily dispersed by ultrasound treatment. Experimentally, MnO_2 precipitates were sonicated at 100 W for a certain period of time to yield homogeneous colloidal solutions of various concentrations (Fig. S3).

Elemental analysis of MnO_2 nanosheets shows that the material is indeed organic-free, exhibiting no potential organic elements, such as C and S, whereas only trace amount (less than 1.0 wt% in total) of Na and K, representing inherent balancing cations, has been detected in addition to Mn and H. Base on the elemental analysis, it is more reasonable to name our 2D MnO_2 nanosheets as $Na_{0.027}K_{0.01}MnO_2$ 0.95 H_2O. However, since such materials are often referred to as MnO_2 in the related literatures (*Chem. Sci.*, 2012, **3**, 433; *J. Am. Chem. Soc.*,

2008, **130**, 15938; *Angew. Chem. Int. Ed.*, 2007, **46**, 4951; *Chem. Commun.*, 2014, **50**, 7885), we followed the tradition to call it MnO_2 in the manuscript for simplification.

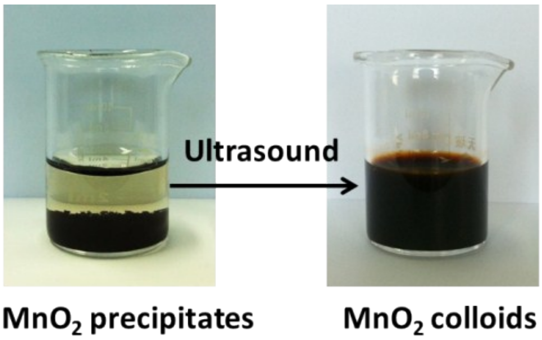

Fig. S3 Photographs of the ultrasound exfoliation to obtain monodispersed MnO_2 nanosheet colloid (right picture) from MnO_2 nanosheet precipitates (left picture).

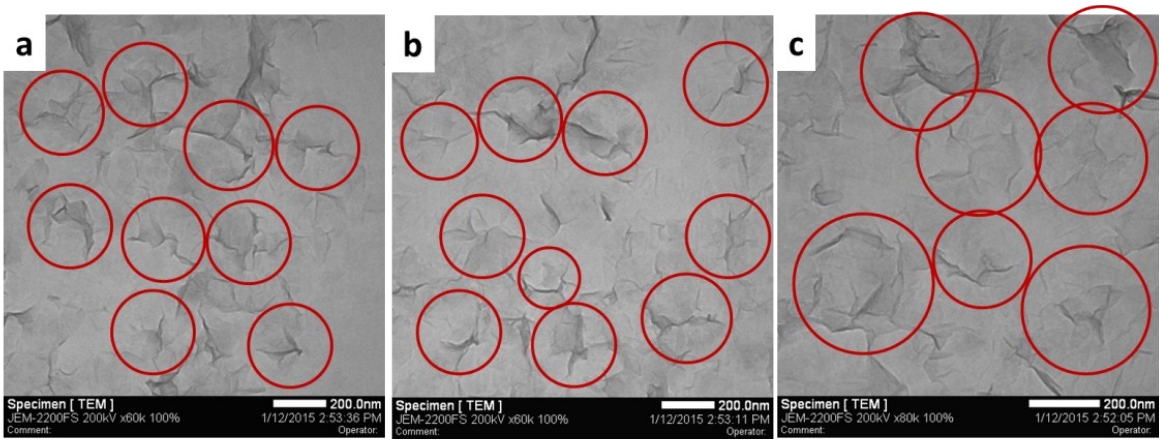

Fig. S4 TEM images of colloidal MnO_2 nanosheets.

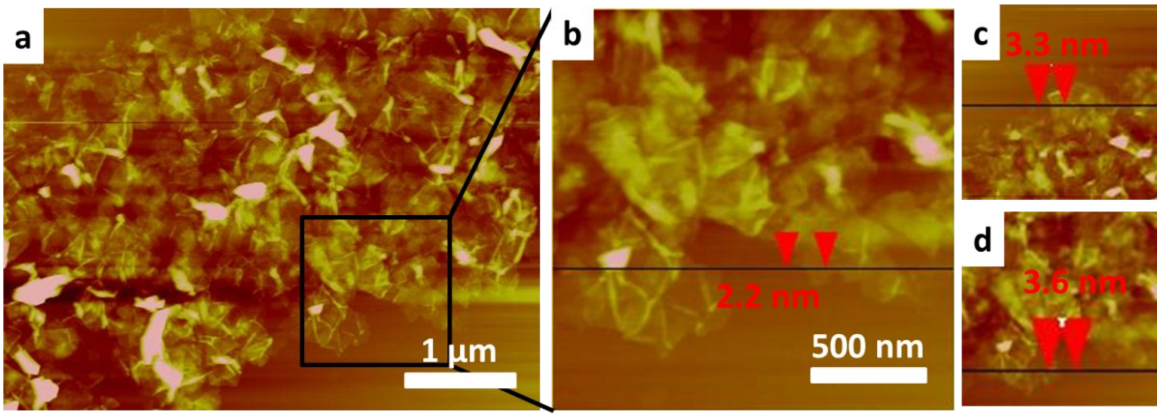

Fig. S5 AFM images of colloidal MnO_2 nanosheets.

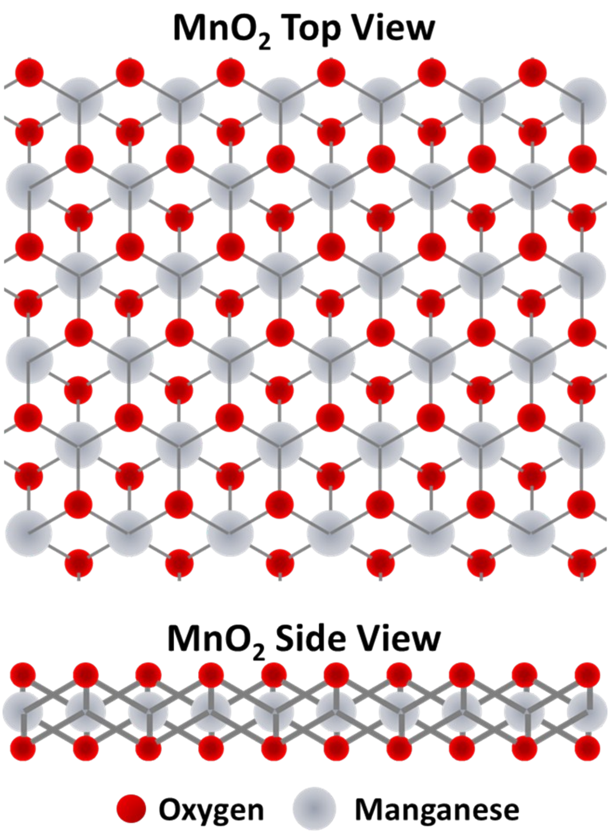

Fig. S6 Chemical structure of the δ-MnO_2 nanosheet. The δ-MnO_2 contains multiple two-dimensional sheets of edge-shared [MnO_6] octahedra. Oxygen atoms are aligned on both sides of the sheets, whereas the layer of Mn atoms is sandwiched between two layers of oxygen atoms. As a result, the negative charge of oxygen atom is exposed on the surface of MnO_2 nanosheets, with the positive charge of Mn being shielded.

S2.3 Preparation of MnO_2 aerogels and density measurement of the aerogel

Colloids of MnO_2 nanosheets (at different concentrations) were poured into a glass mold, which were then frozen at -20 °C for 12 hours to form icy chunks. Subsequently, the obtained frozen samples were directly transferred to a freeze-drier and freeze-dried at -50 °C for 24 hours to prepare free-standing MnO_2 aerogels.

The density (ρ) of the aerogel was determined by dividing the mass weight (m) by the volume (V) (*i.e.* $\rho = m/V$) as done by other researchers (*Adv. Mater.*, 2013, **25**, 2554; *Carbon*, 2014, **80**, 174; *Adv. Mater.*, 2012, **24**, 3486; *Angew. Chem. Int. Ed.*, 2012, **51**, 11371). The lowest density was calculated for the aerogel shown in the inset of Fig. 3c, which maintained a relatively good cylindrical shape after aerogel formation. The minor deformation in the

middle of the aerogel, as shown in the picture, was caused by the forceps used to handle the aerogel in weight measurement. The diameters for the widest and narrowest parts of the deformed aerogel were measured as 1.626 cm and 1.398 cm respectively, and averaged as 1.512 cm. Meanwhile, the height of aerogel (h) was measured as 1.784 cm. The diameters and height were measured using a Vernier caliper (specific to the dividing of 0.02 mm). Subsequently, the volume of the aerogel monolith was calculated based on the geometric equation ($V = \pi (d/2)^2 h$) for cylinder by using the average diameter (d) of 1.512 cm and h of 1.784 cm, which yielded 3.203 cm^3. The weight of the aerogel was measured by an electronic scale (Mettler Toledo, specific to the dividing of 0.0001 g, Fig. S12), which was 1.7 mg. Thus the density (ρ) was determined as 0.53 mg/cm^3.

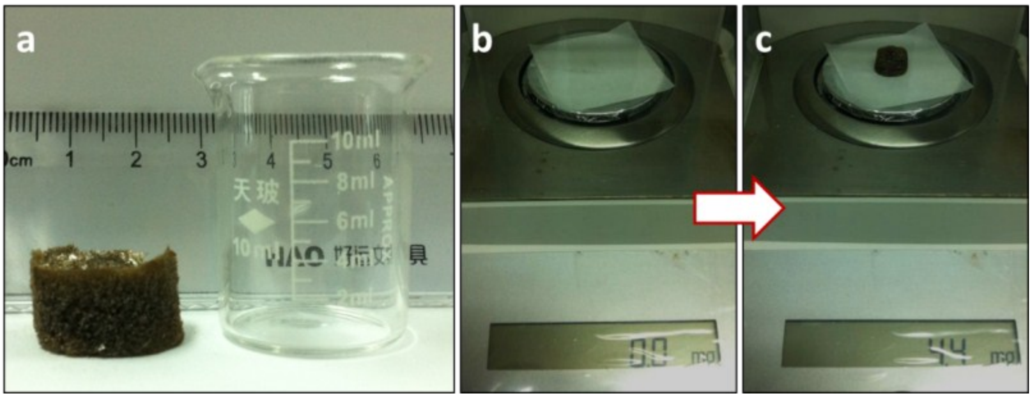

Fig. S7 a) photograph of the ultralight MnO_2 aerogel and the glass mold with a ruler; b-c) the weight measurement of the ultralight MnO_2 aerogel showing a density (ρ) estimated as 1.0 mg cm^{-3} (*i.e.* 4.4 mg in 4.4 cm^3).

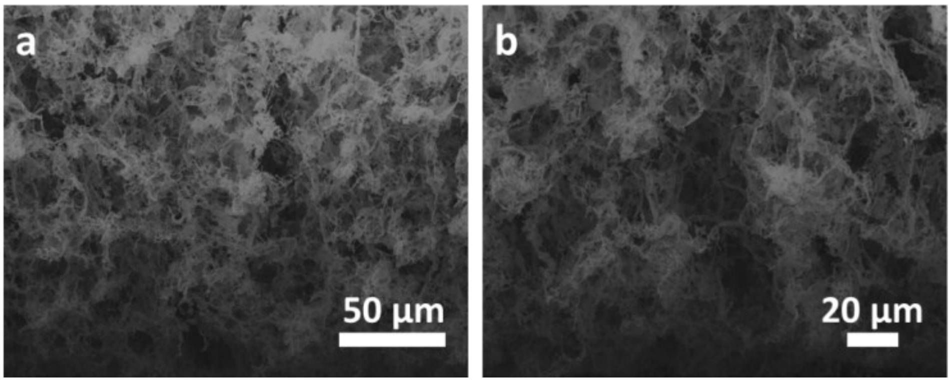

Fig. S8 Sectional-view SEM images of the MnO_2 aerogel (ρ =1.0 mg cm^{-3}) at low (a) and high (b) magnifications.

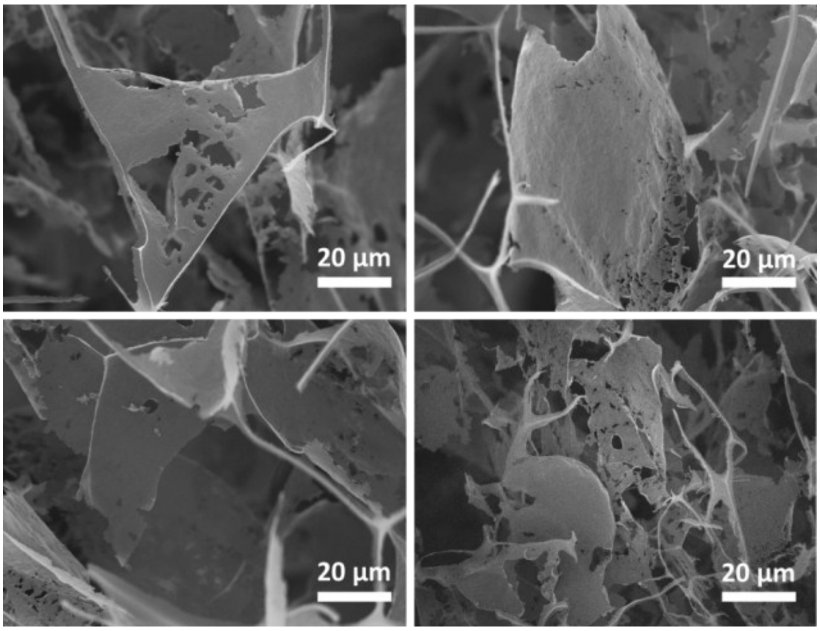

Fig. S9 SEM images of the 2D fakes in the MnO_2 aerogel (ρ =1.0 mg cm^{-3}).

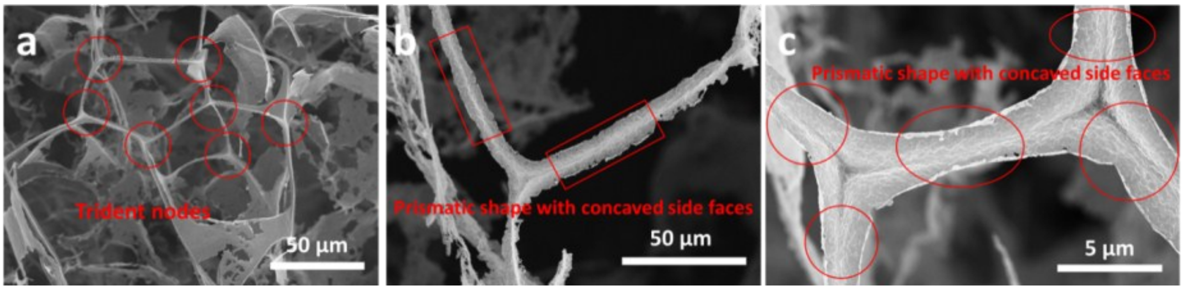

Fig. S10 a) SEM image showing “Y-shaped” trident nodes (marked in red circles); b-c) SEM images of 1D rods displaying a prismatic shape with concaved sides as marked in red boxes (b) and circles (c).

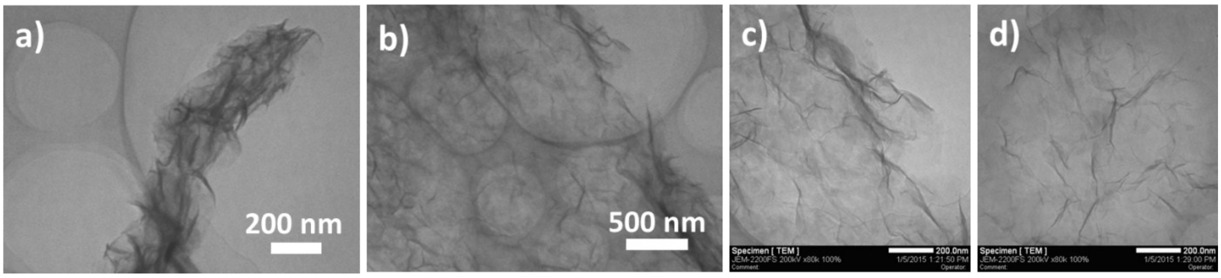

Fig. S11 TEM images of the MnO_2 aerogels: a) a 1D rod; b-d) a 2D flake.

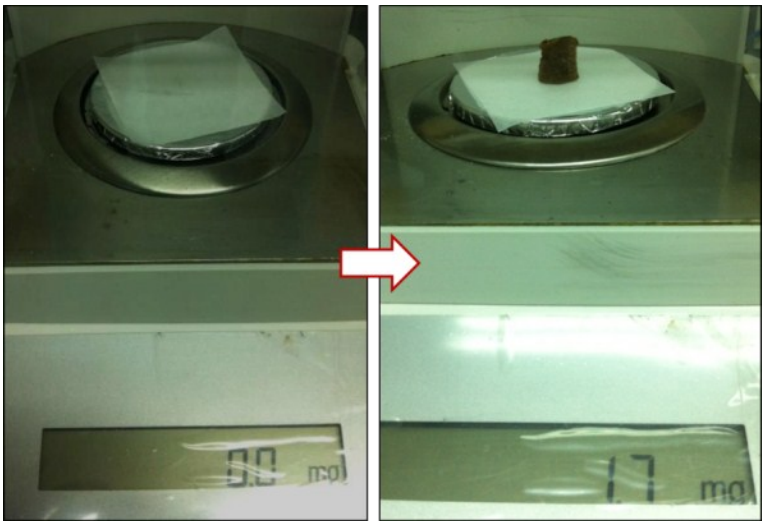

Fig. S12 The weight measurement of the ultralight MnO_2 aerogel prepared from the colloid of 0.5 mg mL^{-1}, showing a density (ρ) estimated as 0.51 mg cm^{-3} (*i.e.* 1.7 mg in 3.34 cm^3).

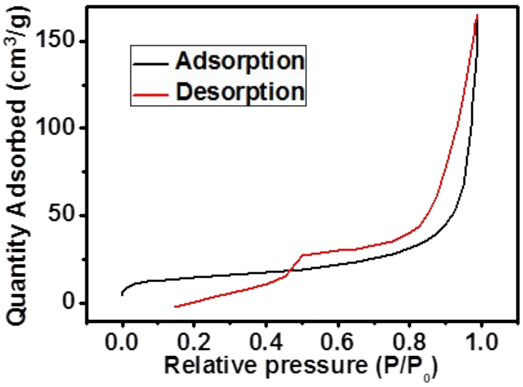

Fig. S13 N_2 Adsorption isotherm of ultrathin MnO_2 nanosheets at a degas temperature of 300 °C.

S3. Freeze-thawed experiment

Aqueous MnO_2 nanosheet colloid (1.0 mg mL^{-1}) was cultivated at -20 °C until all liquid had solidified into a brown ice-containing chunk and then thawed at room temperature. The freeze-thawed sample shows colorless liquid with a large amount of brown sediments at the bottom (Fig. S9). The sediments were dried on silica substrates overnight at room temperature for SEM characterization (Fig. S10). When treated with ultrasound (100 W) for 30 minutes, the freeze-thawed sediments cannot be redispersed into a colloid (Fig. S9), indicating the formation of a stable assembly.

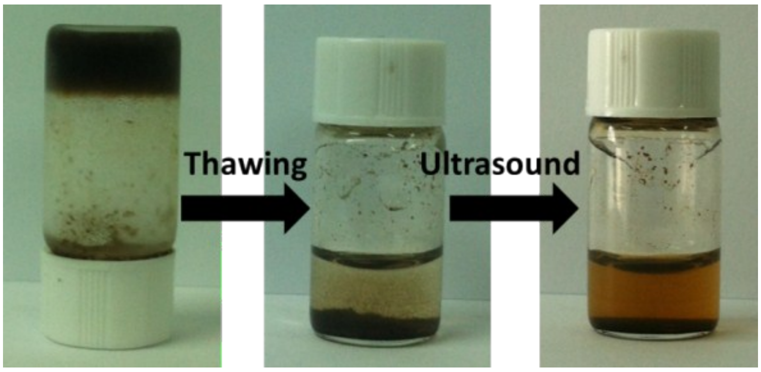

Fig. S14 Photographs of the freeze-thawed process and the ultrasound treatment. Aqueous MnO_2 nanosheet colloid (1.0 mg mL^{-1}) was cultivated at -20 °C to form a brown icy chunk (left picture) and then thawed to yield a colorless liquid with brown sediments (middle picture). After the ultrasound treatment of 100 W for 30 minutes, the majority of the sediments remain undispersed (right picture).

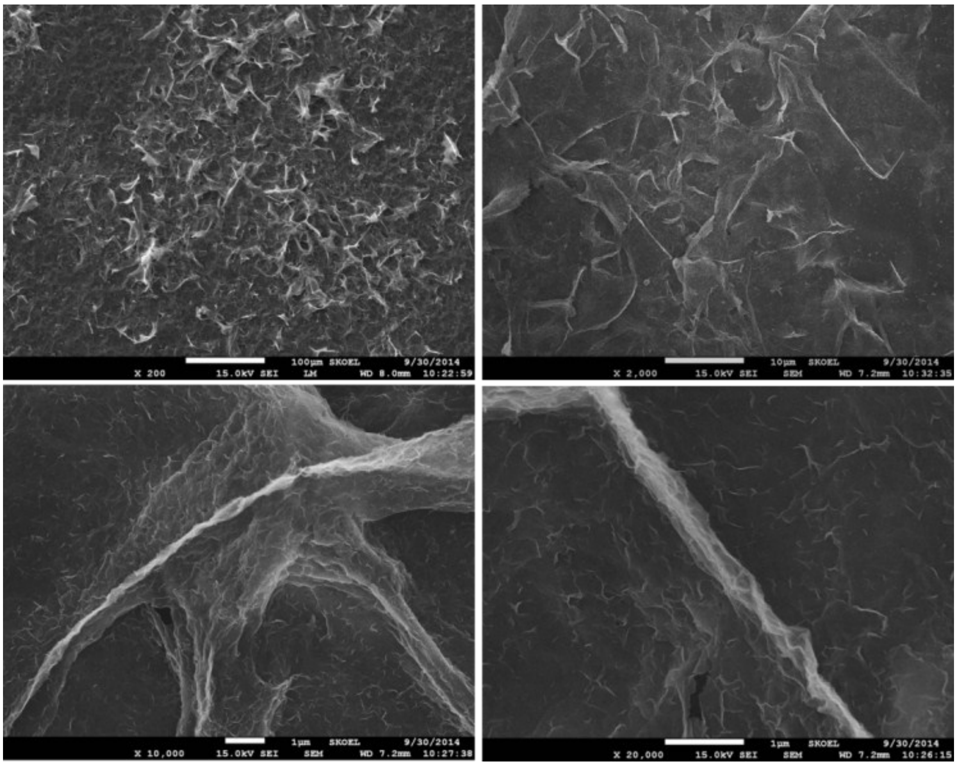

Fig. S15 SEM images of the freeze-thawed samples at different magnifications.

S4. Control experiment using MnO_2 nanospheres as the building blocks

S4.1 Preparation of homogeneous MnO_2 nanosphere colloid

Homogeneous colloid of MnO_2 nanospheres was synthesized by following the procedure described by Xiaoliu Huangfu *et al*. by mixing the appropriate amounts of $KMnO_4$ and $Na_2S_2O_3$ stock solutions (*Environ. Sci. Technol*. 2013, **47**, 10285), according to the following

reaction stoichiometry: $3S_2O_3^{2-}+8MnO_4^{-}+2H^{+}\rightarrow 8MnO_2+6SO_4^{2-}+H_2O$. Briefly, $KMnO_4$ solution was rapidly stirred with a magnetic stir bar, and purged with N_2. Then, the stoichiometric amount of $Na_2S_2O_3$ solution was added drop-wise. The brown MnO_2 nanosphere colloid was formed immediately but stirred for 12 hours to achieve a stable colloidal solution. The stock colloid was stored in the dark at 4°C.

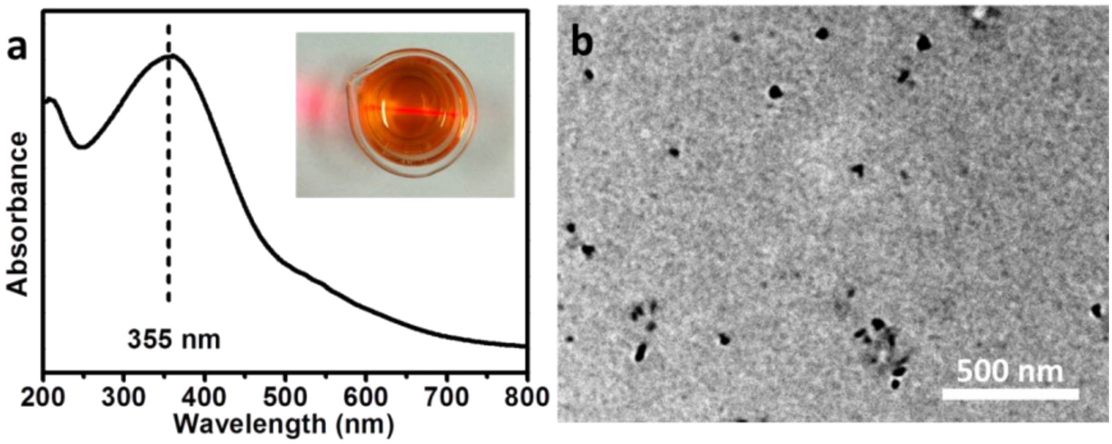

Fig. S16 a) UV-vis spectrum of the MnO_2 nanosphere colloid and photograph of the Tynall phenomenon (inset); b) TEM image of the MnO_2 nanospheres.

S4.2 Construction of aerogels using MnO_2 nanospheres as the building blocks

The control experiment was carried out by using MnO_2 nanospheres instead of 2D MnO_2 nanosheets as the building blocks to construct aerogels under otherwise identical conditions. In details, the colloid of MnO_2 nanospheres (1.0 mg mL^{-1}) was poured into a glass mold, which was then frozen at -20 °C for 12 hours to form icy chunks. Subsequently, the obtained frozen samples were directly transferred to a freeze-drier and freeze-dried at -50 °C for 24 hours.

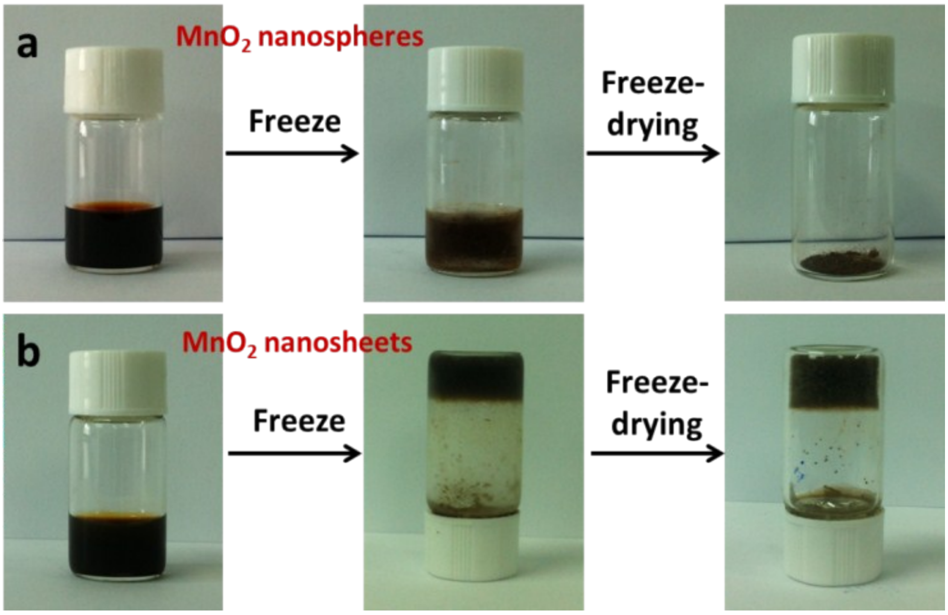

Fig. S17 Photographs of the aerogel construction experiments by using MnO_2 nanospheres (a) and MnO_2 nanosheets (b) as the building blocks under the same conditions. The colloidal solutions (left pictures in (a) and (b)) were frozen at -20 °C to form icy chunks (middle pictures in (a) and (b)) and then freeze-dried to remove ice. The obtained results are shown in the right pictures in (a) and (b).

S5. MnO_2 aerogels as the absorbents for hydrazine

To produce hydrazine vapor, a small droplet (1 μL) of $N_2H_4 \cdot H_2O$ was dropped at the bottom of a conical flask and heated at 60 °C for 10 minutes to evaporate. For the experiment in the absence of absorbents, the sealed N_2H_4 vapor was collected by a syringe and injected into the N_2H_4-sensitive colorimetric probe (5 μg/mL colloidal suspension of MnO_2 nanosheets). For the experiment in the presence of absorbents, two pieces of MnO_2 aerogels (3.0 mg for each) or commercial MnO_2 powders (6.0 mg) were used as the absorbents in the conical flask as N_2H_4 vapor being produced, and the residual N_2H_4 vapor was collected by a syringe and injected into the colorimetric probe for detection.
